# P-228. Prevalence of Fraility and Pre-fraility in virally Suppressed Persons Living With HIV on Antiretroviral Therapy

**DOI:** 10.1093/ofid/ofaf695.450

**Published:** 2026-01-11

**Authors:** Jayjit Guha, A T U L GOGIA, Richa Dewan, Atul Kakar, Shristi Paul Byotra, Rishikesh Dessai

**Affiliations:** Sir Ganga Ram Hospital, New delhi, Delhi, India; Sir Ganga Ram Hospital, New Delhi, India, NEW DELHI, Delhi, India; Sir Ganga Ram Hospital, New delhi, Delhi, India; Sir Ganga Ram Hospital, New delhi, Delhi, India; Sir Ganga Ram Hospital, New delhi, Delhi, India; Sir Ganga Ram Hospital, New delhi, Delhi, India

## Abstract

**Background:**

With the advent of antiretroviral therapy (ART), persons living with HIV (PLHIV) are achieving successful viral suppression and experiencing increased longevity. However, HIV infection and ART contribute to accelerated aging, manifesting in age-related conditions like cognitive decline, diabetes mellitus, osteoporosis, and cardiovascular disease. Frailty, a key phenotype of aging, is more prevalent in PLHIV than in the general population. This study investigates the prevalence of frailty and pre-frailty in virally suppressed PLHIV on ART in India and explores associated sociodemographic and medical factors.

**Methods:**

The study population included virally suppressed adults living with HIV on ART with viral load < 1000 copies/ml for last 6 months or more. The Physical Frailty Phenotype criteria was used to assess frailty, categorizing participants as frail, pre-frail, or non frail.

**Results:**

A total of 96 participants were enrolled. The mean age was 40.78 years, with 82.3% being male. The mean BMI was 25.44 kg/m². The average duration of HIV infection and ART was 4.74 and 4.66 years, respectively. The mean current CD4 count was 512.04 cells/µL, and all participants were virally suppressed.

Frailty assessment revealed that 3.1% of participants were frail, 44.8% were pre-frail, and 52.1% were non-frail. Among the parameters checked to assess frailty, weakness (71.7%) and low activity level (43.4%) were the most affected parameters. Age was significantly associated with frailty, with a higher prevalence in participants aged 50 years and above. Gender, socioeconomic status, BMI, Duration of HIV infection and ART, type of ART regimen, CD4 count, comorbidities, past history of opportunistic infection and degree of cognitive impairment were not significantly associated with frailty and pre-frailty

**Conclusion:**

Pre-frailty was more prevalent than frailty among virally suppressed PLHIV with higher prevalence in those aged 50 and above. Notably, many under 50 showed high prevalence of pre-frailty, indicating its earlier onset in PLHIV. The findings highlight the vulnerability of PLHIV to pre-frailty and frailty across all ages, emphasizing the need for early diagnosis and intervention planning.

**Disclosures:**

All Authors: No reported disclosures

